# BAP1 functions as a tumor promoter in prostate cancer cells through EMT regulation

**DOI:** 10.1590/1678-4685-GMB-2019-0328

**Published:** 2020-05-08

**Authors:** Chan Mi Park, Jae Eun Lee, Jung Hwa Kim

**Affiliations:** 1 Department of Biological Sciences, Inha University, Incheon 22212, South Korea

**Keywords:** BAP1, EMT, prostate cancer cells

## Abstract

BRCA1-associated protein 1 (BAP1) is a deubiquitinating enzyme that has long been considered to be a tumor suppressor in various tumors, including renal cell carcinoma, uveal melanoma, mesothelioma, and cutaneous melanoma. However, the involvement of BAP1 in the progression of prostate cancer has not been studied until recently. Herein, we investigated the tumor promoting function of BAP1 in the context of prostate cancer. Analysis of The Cancer Genome Atlas (TCGA) data set showed that prostate cancer patients express high levels of BAP1 mRNA. High BAP1 expression is inversely correlated with disease-free survival in patients with prostate cancer. Among the prostate cell lines tested, BAP1 expression was high in tumorigenic and metastatic cell lines, but was low in normal prostate cell line. Knockdown of BAP1 in PC3 or DU145 cells induced mesenchymal-to-epithelial transition (MET). Further, BAP1-knockdown resulted in decreased migration and invasion of PC3 and DU145 cells. Conversely, overexpression of BAP1 in RWPE1, a normal prostate cell line, induced the migratory and invasive properties. Collectively, our findings identified that BAP1 has a tumor promoting function in prostate cancer cells, and suggest that BAP1 can serve as a potential therapeutic target for prostate cancer.

## Introduction

BRCA1-associated protein 1 (BAP1) is a deubiquitinating enzyme, which cleaves ubiquitin (Ub) from substrate proteins, and is subcategorized into ubiquitin C-terminal hydrolases (UCHs) (Fang and Shen, 2017). BAP1 was originally identified as a novel binding protein, which interacted with the tumor suppressor BRCA1 (Jensen *et al.*, 1998). Recent investigations suggest that BAP1 functions in various biological processes, including the DNA damage response, transcriptional regulation, chromatin dynamics, and cell cycle regulation. BAP1 is known to assemble multiprotein complexes with numerous cofactors and transcription factors including Ying Yang 1 (YY1) and host cell factor 1 (HCF1) (Yu *et al.*, 2010). Furthermore, ternary complexes of BAP1 with the forkhead transcription factors FoxK1/K2 and HCF1 have been reported. The catalytic activity of BAP1 is attributed to its repression of FoxK2 target genes (Okino *et al.*, 2015). BRCA1/BARD1 is a RING heterodimer E3 ligase that is involved in the regulation of DNA damage response. BAP1 binding to BARD1 results in inhibition of the E3 ligase activity of BRCA1/BARD1 via the prevention of RING heterodimer formation (Nishikawa *et al.*, 2009). Polycomb repressive complexes (PRCs) silence gene expression via histone modifications. Trimethylation of the histone H3 at lysine 27 (H3K27me3) by PRC2 triggers the recruitment of PRC1 and subsequent ubiquitination of histone H2A at lysine 119 (H2AK119ub) by PRC1, which fixes chromatin in a repressed state and silences gene expression (Murali *et al.*, 2013). BAP1 protein forms a complex with ASXL1/2 to give rise to the polycomb repressive deubiquitinase complex (PR-DUB) that deubiquitinates H2AK119ub and reverses H2A ubiquitination-mediated gene repression (Scheuermann *et al.*, 2010).

A plethora of evidences from genetic studies has demonstrated that BAP1 could suppress tumorigenesis. Germline *BAP1* mutations are associated with the development of several tumors including renal cell carcinoma, mesothelioma, uveal melanoma, and various other malignancies. Somatic *BAP1* mutations are frequently associated with various tumors, including metastatic uveal melanomas, small cell lung carcinoma, and malignant mesotheliomas (Carbone *et al.*, 2013; Cheung and Testa, 2017). However, BAP1 is rarely mutated in prostate cancers (Je *et al.*, 2012). Importantly, the involvement of BAP1 in the progression of prostate cancer has not been studied until recently.

On analyzing the data hosted on The Cancer Genome Atlas (TCGA) database, we found that the expression of BAP1 mRNA is increased in the patients with prostate cancer, and that high BAP1 expression is inversely correlated with disease-free survival. Furthermore, we identified that BAP1-knockdown results in the inhibition of migration/invasion and induction of MET in prostate cancer cells. Our findings indicate that BAP1 functions as a tumor promoter and could serve as a potential target for preventing prostate cancer.

## Material and Methods

### TCGA data analysis

To analyze the expression of BAP1 mRNA in normal and cancer samples, Level3 data (RNA-seq V2) describing mRNA expression and clinical and survival data were downloaded from the TCGA (http://cancergenome.nih.gov/). The Level3 data comprised normal data (n = 52), metastasis data (n = 1), and cancer data (n = 497). To verify the difference in mRNA expression, the mRNA levels of each sample were converted into logarithm to the base of 2. The statistical difference in the expression of BAP1 mRNA between groups was assessed using Wilcoxon rank sum test that was processed using the Wilcox test package of R. The results were represented using box-plot. To compare the expression of BAP1 mRNA in primary and metastatic cancer patients, we used the publicly available cBioPortal platform (http://www.cBioportal.org). Raw data is available in NCBI GEO under the accession no GSE21302. The analyzed data obtained via the cBioPortal was composed of primary tumor (n = 131) and metastatic tumor (n = 19) samples. Statistical differences were examined by two-tailed Student’s *t*-tests and all statistical analyses were performed by R software.

### Survival analysis

To determine the correlation between BAP1 expression and disease-free survival, the TCGA-PRAD clinical data were downloaded from cBioPortal. RNA seq data and disease-free survival data was available for 491 patients. Patients were divided into high- (n = 246) and low-expression groups (n = 245) using the median expression level of BAP1 mRNA as the cut-off. Survival curves were calculated using Kaplan-Meier analysis and log-rank tests.

### Maintenance of cell lines

DMEM supplemented with 10% FBS was used to culture PC3 cells and RPMI 1640 with 10% FBS was used to culture DU145 cells. Keratinocyte serum-free medium containing 1.25 μg/l EGF and 12.5 mg/l bovine pituitary extract was used to culture RWPE1 cells. An antibiotic-antimycotic solution was added to all media. All cells were grown in incubator set at 37 °C and containing a 5% CO_2_ atmosphere.

### BAP1 C91S mutant construction

The nPfu-Forte DNA polymerase was used to construct a BAP1 C91S mutant plasmid by site-directed mutagenesis. The following primers were used: 5’-CAG CTG ATA CCC AAC TCT AGT GCA ACT CAT GCC TTG CTG-3’ and 5’-CAG CAA GGC ATG AGT TGC ACT AGA GTT GGG TAT CAG CTG-3’. The mutation was confirmed by DNA sequencing.

### Antibodies and western blotting

The following antibodies were used in this study: anti-BAP1 (sc-28383, Santa Cruz), anti-vimentin (sc-32322, Santa Cruz), anti-E-cadherin (610181, BD Transduction Laboratories), anti-Flag-M2 (F3165, Sigma-Aldrich), and anti-β-actin (A1978, Sigma-Aldrich). For immunoblot assay, protein samples were subjected to SDS-PAGE and transferred onto PVDF membranes. After incubation with the appropriate primary antibody and corresponding HRP-conjugated secondary antibody, protein bands were detected with enhanced chemiluminescence solution.

### Construction of BAP1-knockdown stable cell lines

The target sequences used for small hairpin RNA (shRNA) against BAP1 were 5’-CCAACTCTTGTGCAA CTCA-3’ (shRNA1) (Qin *et al.*, 2015) and 5’-GGAGGA GATCTACGACCTTCA-3’ (shRNA2). Annealed BAP1 shRNA primers were ligated into pMSCVpuro vector. To generate retroviruses, the cloned pMSCVpuro-shBAP1 plasmid, MLV, and VSV-G vectors were co-transfected into HEK293 cell lines using Lipofectamine plus (Invitrogen) transfection reagent. After 48 h, the retroviruses were collected. Filtered retroviruses were transduced into PC3 or DU145 cells with polybrene. For selection, cells were maintained with puromycin (5 μg/ml) 48 h after infection. After 2 weeks, stable BAP1 shRNA transfectants were selected. In knockdown assays, pMSCVpuro empty vector was used as control and designated as MSCV.

### Real-time RT-PCR

TRIzol was used for total RNA extraction. Oligo (dT) primers and RevertAid reverse transcriptase were used for reverse transcription. In addition, real-time quantitative RT-PCR was performed to detect the relative mRNA levels using the SYBR Green and ABI prism 7300 system. The ΔΔCt method was used for the calculation of mRNA level of each gene, and *GAPDH* was used as a reference. The following primer pairs were used in this study: *BAP1* 5’-CGATCCATTTGAACAGGAAGA-3’ and 5’-CTCGT GGAAGATTTCGGTGT-3’; E-cadherin 5’-GTCACTGAC ACCAACGATAATCCT-3’ and 5’-TTTCAGTGTGGTG ATTACGACGTTA-3’ (Ye *et al.*, 2010); *Vimentin* 5’-CT CCACGAAGAGGAAATCCA-3’ and 5’-GGTCAGCA AACTTGGATTTGTA-3’ (Elloul *et al.*, 2010); *Twist* 5’- GGAGTCCGCAGTCTTACGAG-3’ and 5’-TCTGG AGGACCTGGTAGAGG-3’ (Yang *et al.*, 2004); *Snail* 5’- TTCTCTAGGCCCTGGCTGCTACAA-3’ and 5’-TC TTGACATCTGAGTGGGTCTGGA-3’ (Ye *et al.*, 2010); *MMP2* 5’-CTTCTTCAAGGACCGGTTCAT-3’ and 5’-GC TGGCTGAGTAGATCCAGTA-3’ (Miyoshi *et al.*, 2005); *MMP7* 5’-CACTGTTCCTCCACTCCATTTAG-3’ and 5’-CATTTATTGACATCTACCCACTGC-3’, *MMP9* 5’-AA AACCTCCAACCTCACGGA-3’ and 5’-GCGGTACAAG TATGCCTCTGC-3’ (Yen *et al.*, 2010), and *VEGFA* 5’- AGACTCCGGCGGAAGCAT-3’ and 5’-AATGGCGAATCCAATTCCAA-3’ (Kaushal *et al.*, 2005). All experiments were performed in triplicates.

### Cell Proliferation assay

PC3 (5 × 10^4^), DU145 (5 × 10^4^) or RWPE1 (2 × 10^4^) cells were seeded in 6-well plates in duplicate at day 0 (D0) and cell viability was determined at D1, D2, D3, and D4. After staining with trypan blue solution, the number of viable cells were counted using a hemocytometer under a microscope.

### Wound healing assay

In this assay, PC3, DU145 or RWPE1 cells (2 × 10^5^) were seeded into six-well plates. When cells reached 95-100% confluence, sterile micropipette tips were used to create a denuded area. After removing the detached cells by washing with PBS, the cells were supplemented with serum-free medium. The samples were photographed using a light microscope (IX51, Olympus) at 50× magnification. The percentage of wound closure area at 17 h (PC3 and DU145) or 20 h (RWPE1) was analyzed using the ImageJ software.

### Transwell cell migration and invasion assay

Transwell chambers with 8 μm pore size polycarbonate membrane filters (Corning) were used to analyze cell migration and invasion. The membrane was pre-coated with Matrigel (Corning) for invasion, but not for migration assays. A total of 1 × 10^4^ cells for migration and 2 × 10^4^ cells for invasion assays were suspended in serum-free medium and seeded in the upper chamber, and the lower chamber was filled with medium containing 15% FBS. After incubation at 37 °C for 22 h, the cells on the upper surface of the filter were removed. Cells that had migrated or invaded the bottom surface were fixed with 100% methanol and stained with 0.5% Giemsa solution. The number of cells were counted under a light microscope.

### Immunofluorescence

PC3 or DU145 cells were seeded on PLL-coated glass coverslips. After fixing with 2% formaldehyde in PBS for 30 min, the cells were permeabilized with PBS containing 0.5% Triton X-100. After rinsing with PBS containing 0.1% Triton X-100 (PBST), the cells were incubated in PBST containing 3% horse serum and 10% gelatin for 30 min. The cells were then incubated with E-cadherin or vimentin antibody overnight at 4 °C and washed with PBST. After incubation with fluorescein isothiocyanate-conjugated secondary antibody (Jackson laboratories) for 1 h, the cells were rinsed with PBST. The coverslips were mounted with Vectashield containing DAP1 (Vector Laboratories) and cells were visualized with a Zeiss Axiovision/LSM 510 META inverted confocal microscope.

## Results

### High BAP1 expression is inversely correlated with disease-free survival in prostate cancer patients

**Figure 1:**
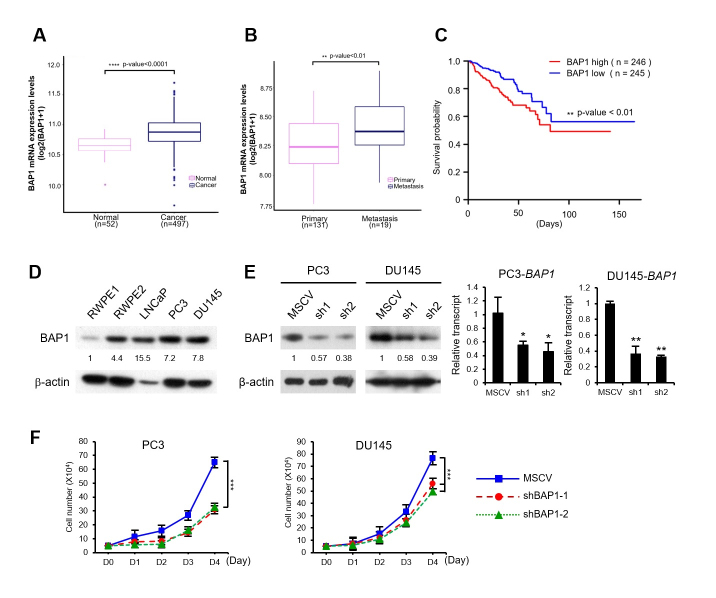
High BAP1 expression is inversely correlated with disease-free survival in prostate cancer patients. (A) The expression of BAP1 mRNA in normal samples (n = 52) and cancer samples (n = 497) was compared using box-plot. The data were downloaded from the TCGA-PRAD. Significance was assessed by Wilcoxon rank sum test (p < 0.0001). (B) The expression of BAP1 mRNA in primary tumors (n = 131) and metastatic tumors (n = 19) was compared. Raw data is available in NCBI GEO under the accession no GSE21302. The analyzed data was obtained via the cBioPortal. Statistical differences were examined by two tailed Student’s *t*-test (p < 0.01). All statistical analyses were performed using R software. (C) Analysis of disease-free survival in the TCGA-PRAD dataset. Kaplan-Meier survival curves for high (n = 246) and low (n = 245) BAP1 expression groups. Each group was separated by the median expression of BAP1. (D) The expression of BAP1 in various prostate cancer cells was analyzed by western blotting. The band density was quantified by densitometry. (E) Generation of stable BAP1-knockdown cell lines using shBAP1. Western blot and real-time RT-PCR analysis of stable BAP1-knockdown cell lines. The band density was quantified by densitometry. (F) Cell growth curves of BAP1-knockdown cell lines. Viable cells were counted by trypan blue-exclusion assay every 24 h after cell seeding. The *p* values were calculated by Student’s *t*-test. ***p < 0.001

To address the BAP1 function with respect to the progression of prostate cancer, we compared the mRNA levels of BAP1 in normal prostate samples (n = 52) and prostate cancer samples (n = 497) using data from the TCGA dataset. BAP1 expression was significantly higher in prostate carcinoma tissues than that in the normal tissues (*p* < 0.0001, Wilcoxon rank sum test) (Figure 1A). To further validate the involvement of BAP1 in prostate cancer, we compared BAP1 expression in primary and metastatic prostate cancer using the publicly available cBioPortal platform (http://cBioportal.org). Raw data is available in NCBI GEO under the accession no GSE21302. The analyzed data obtained via the cBioPortal consists of 131 primary and 19 metastatic prostate cancer samples. BAP1 was expressed at higher levels in metastatic cancer samples compared with that in primary tumor samples (*p* < 0.01, Student’s *t*-test) (Figure 1B). Next, we investigated whether the disease-free survival of prostate cancer patients is associated with BAP1 expression level. Kaplan-Meier analysis of a total of 492 patients with prostate cancer showed that patients with high BAP1 expression had worse disease-free survival compared with that of patients with relatively low BAP1 expression (*p* < 0.01, log-rank test) (Figure 1C). Thus, results from the TCGA cohort studies suggest that BAP1 might have a tumor promoting ability during the progression of prostate cancer.

In order to investigate the positive role of BAP1 in the development of prostate cancer, we assessed the BAP1 levels in several prostate cancer cell lines. Notably, the expression of BAP1 protein was high in tumorigenic and metastatic cell lines, including RWPE2, LNCaP, PC3, and DU145, but was low in normal prostate cell line RWPE1 (Figure 1D). Then, to characterize BAP1 function in the development of prostate cancer, we established a stable BAP1-knockdown in PC3 and DU145 cell lines using BAP1 specific shRNAs (Figure 1E). The proliferation of BAP1-knockdown cells was inhibited relative to that of the control (Figure 1F). These results suggest a possible tumor promoting function for BAP1 in prostate cancer cells. To investigate whether the inhibition of cell growth—resulting from BAP1 depletion—is involved in cell death, we checked the expression of several pro-apoptotic and anti-apoptotic genes in BAP1-knockdown stable cells. The expression of both pro-apoptotic (*BAX*, *BIK*, and *BAD*) and anti-apoptotic genes (*MCL-1* and *BCL-XL*) was not affected by BAP1-knockdown in prostate cancer cells (Figure S1, Table S1).

### BAP1-knockdown induces MET in prostate cancer cells

**Figure 2:**
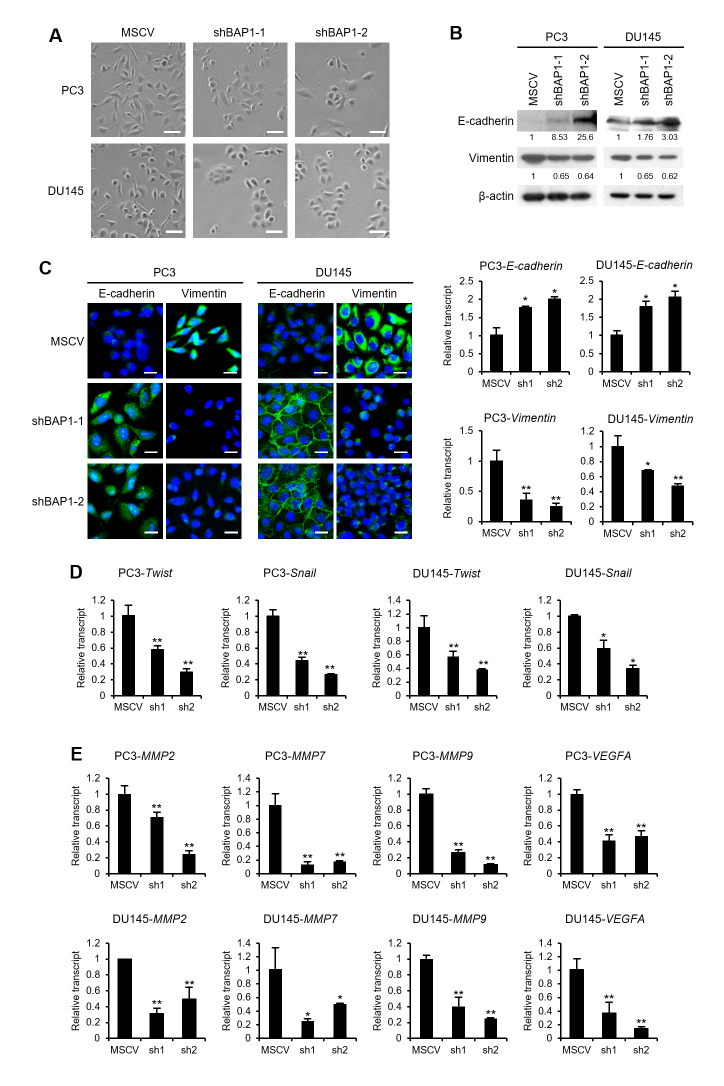
BAP1-knockdown induces MET in prostate cancer cells. (A) Images of PC3 or DU145 cells with BAP1-knockdown (100 magnification). Scale bar represents 100 μm. (B) Immunoblotting and real-time RT-PCR for E-cadherin and vimentin in BAP1-knockdown cell lines. The band density was quantified by densitometry. (C) Immunofluorescence for E-cadherin or vimentin (green) in BAP1-knockdown cells. Nuclear DNA was stained with DAPI and the image was merged with that of E-cadherin or vimentin. Scar bar represents 20 μm. (D) Real-time quantitative RT-PCR for *Twist* and *Snail* mRNAs in BAP1-knockdown cells. Values are expressed as mean ± SD of three independent experiments. (E) Real-time quantitative RT-PCR for *MMP2*, *MMP7*, *MMP9*, and *VEGFA* mRNAs in BAP1-knockdown cells. Values are expressed as mean ± SD of three independent experiments. The *p* values were calculated using Student’s *t*-test. *p < 0.05, **p < 0.01

Tumor cells acquire invasive and metastatic abilities by undergoing dynamic processes such as EMT, which are accompanied by morphological and molecular changes. To investigate the involvement of BAP1 in EMT, we examined the effect of BAP-knockdown on cell morphology. BAP1-knockdown cells became less elongated and more tightly packed compared to the control cells (Figure 2A); this morphological change was attributed to MET, a process that is the reverse of EMT. To confirm the induction of MET upon BAP1 depletion, we examined the expression of E-cadherin and vimentin in BAP1-knockdown cells. Knockdown of BAP1 induced the molecular alterations associated with MET, such as increased E-cadherin expression and decreased vimentin expression at protein and mRNA levels (Figure 2B). Furthermore, immunofluorescence confirmed MET induction in response to BAP1-knockdown (Figure 2C). EMT is induced by several transcription factors including Snail and Twist (Yang and Weinberg, 2008). BAP1-knockdown decreased the expression of *Twist* and *Snail* in PC3 and DU145 cells (Figure 2D). Matrix metalloproteases (MMPs), which digest the extracellular matrix, are important players in the invasion and metastasis of tumor cells (Cavallaro and Christofori, 2004). Knockdown of BAP1 also inhibited the expression of *MMP2*, *MMP7*, and *MMP9* (Figure 2E). Further, vascular endothelial growth factor A (VEGFA), which is crucial for angiogenesis, was also downregulated upon BAP1-knockdown. These results indicate that BAP1 is required for EMT in prostate cancer cells.

### BAP1 promotes the migration and invasion of prostate cancer cells

**Figure 3:**
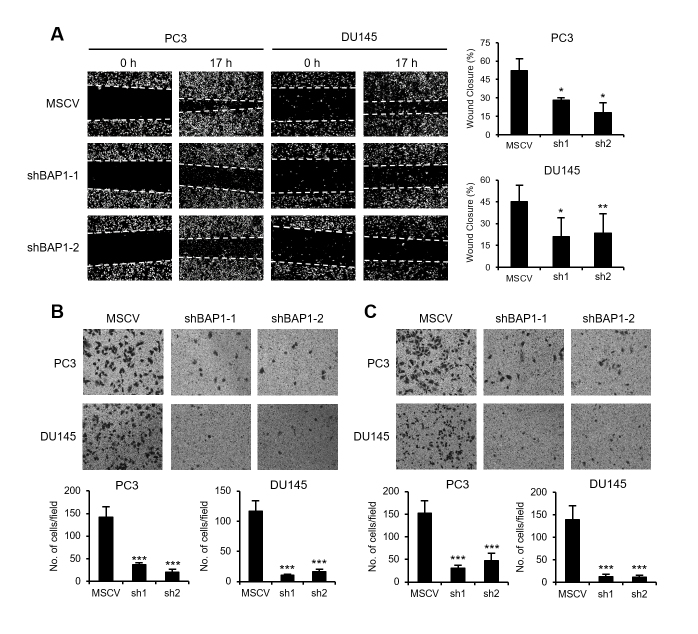
BAP1 promoted the migration and invasion abilities of prostate cancer cells. (A) Wound healing assays on PC3 and DU145 cells with BAP1-knockdown. Values are expressed as the mean ± SD of three independent experiments. (B) Transwell migration assays on PC3 and DU145 cells with BAP1-knockdown. Values are expressed as the mean ± SD of three independent experiments. (C) Matrigel invasion assays on BAP1-knockdown PC3 and DU145 cells. Values are expressed as the mean ± SD of three independent experiments. The *p* values were calculated using Student’s *t*-test. *p < 0.05, **p < 0.01, and ***p < 0.001

In addition, we investigated whether the EMT regulation by BAP1 is implicated in the migration and invasiveness of prostate cancer cells. In BAP1-knockdown background, wound healing ability of the tested cells was decreased (Figure 3A). Transwell migration assays showed that BAP1-knockdown also inhibited the migration ability of prostate cancer cells (Figure 3B). The invasion through the Matrigel was decreased upon BAP1-knockdown (Figure 3C). These results suggest that BAP1 might promote the migration and invasion of prostate cancer cells by inducing EMT.

### Overexpression of BAP1 increases the migration and invasion of RWPE1 cells

**Figure 4:**
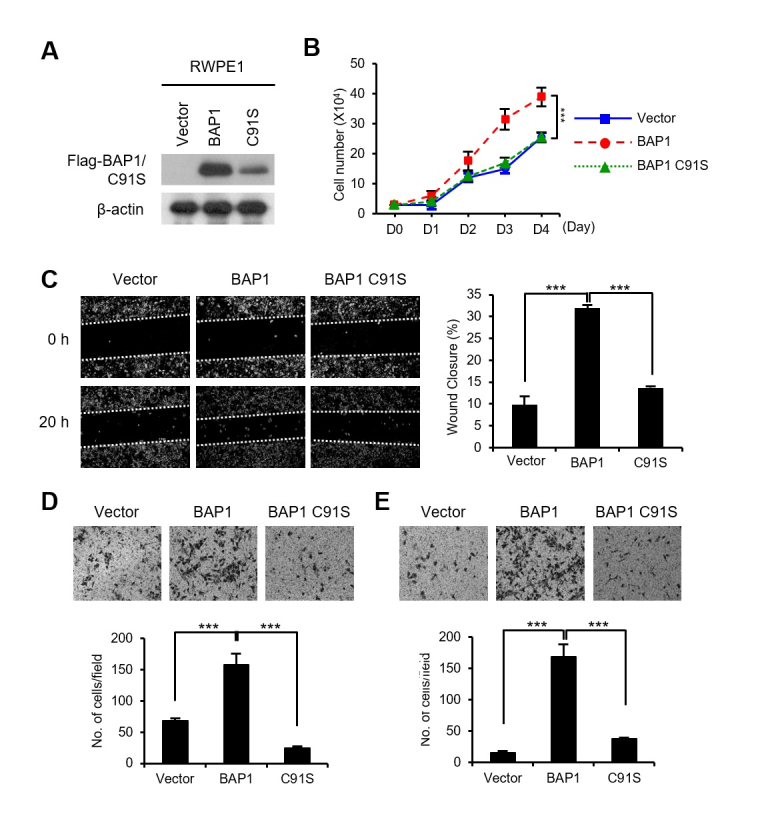
BAP1 increased the migration and invasion of RWPE1 cells. (A) Ectopic expression of Flag-BAP1 or Flag-BAP1 C91S in RWPE1 cells was verified by immunoblotting. (B) Cell growth curves of the BAP1 or BAP1 C91S overexpressing RWPE1 cells. Viable cells were counted by trypan blue-exclusion assay every 24 h after cell seeding. (C) Wound healing assays on BAP1 or BAP1 C91S-overexpressing RWPE1 cells. Values are expressed as the mean ± SD of three independent experiments. (D) Migration assays on BAP1 or BAP1 C91S-overexpressing RWPE1 cells. Values are expressed as the mean ± SD of three independent experiments. (E) Matrigel invasion assays on BAP1 or BAP1 C91S-overexpressing RWPE1 cells. Values are expressed as the mean ± SD of three independent experiments. The *p* values were calculated using Student’s *t*-test. *p < 0.05, **p < 0.01, and ***p < 0.001

We had found that the BAP1 protein was expressed at very low levels in RWPE1, the normal prostate cell line, relative to its expression in tumorigenic and metastatic prostate cancer cells (Figure 1C). Therefore, to further investigate the tumor promoting activity of BAP1 in prostate cancer cells, we introduced BAP1 or the Ub hydrolase activity deficient BAP1 mutant (BAP1 C91S) in RWPE1 cells (Figure 4A). As shown in Figure 4B, BAP1 overexpression increased the proliferation of RWPE1 cells, but BAP1 C91S did not. BAP1 also induced the invasive and migratory properties in RWPE1 cells, as evidenced by wound healing, migration, and invasion assays (Figure 4C-E). However, BAP1 C91S did not induce the invasive and migratory properties in RWPE1 cells. Taken together, we conclude that BAP1 has a tumor promoting activity in prostate cancer cells, which is dependent on its Ub hydrolase activity.

## Discussion

BAP1 is frequently mutated in many human cancers and is widely recognized as a tumor suppressor (Carbone et al., 2013; Wang et al., 2016). However, BAP1 mutation has rarely been identified in prostate cancer (Je et al., 2012). In addition, the involvement of BAP1 in the progression of prostate cancer has not been elucidated yet. In this report, we explored the involvement of BAP1 in the invasion and metastasis of prostate cancer cells through regulation of EMT. On analyzing the TCGA dataset, we found high expression of BAP1 mRNA in patients with prostate cancer. Additionally, high BAP1 expression in patients was associated with low disease-free survival. Interestingly, BAP1 expression was very low in the normal prostate cell line, but was high in tumorigenic and metastatic prostate cell lines. BAP1-knockdown in PC3 or DU145 induced MET by inducing E-cadherin expression and suppressing vimentin expression. This change in the expression of MET-related factors was consistent with the decreased expression of the key EMT-inducing factors including *Twist* and *Snail* in BAP1-knockdown cells. MMPs are known to be involved in EMT and their expression is an essential feature of EMT (Peinado *et al.*, 2007). Knockdown of BAP1 resulted in reduced expression of several MMPs including *MMP2*, *MMP7*, and *MMP9*. Further, the inhibition of EMT upon BAP1 knockdown resulted in a decrease in the proliferation, migration, and invasion of PC3 and DU145 cells. Conversely, RWPE1—a normal prostate cell line—acquired invasive and metastatic properties in response to overexpression of BAP1.

Many data support the inhibitory role of BAP1 in cell proliferation and cancer progression. BAP1 was found to inhibit the cell growth of breast, renal, and lung cancer cells (Jensen *et al.*, 1998; Ventii *et al.*, 2008; Pena-Llopis *et al.*, 2012). Importantly, BAP1 is also known to be positively associated with cancer progression. Depletion of BAP1 in breast cancer cell lines caused growth inhibition that was dependent on its deubiquitinating activity (Machida *et al.*, 2009). Qin *et al*. identified that BAP1 knockdown resulted in inhibition of growth and metastasis of breast cancer cells (Qin *et al.*, 2015). The role of BAP1 in the promotion of myeloid leukaemogenesis has also been reported. (Asada *et al.*, 2018).

Overall, depending on the cell and tissue-type, BAP1 plays diverse roles during tumor development. The function of BAP1 as a tumor suppressor has been identified in many tumors, including renal cell carcinoma, mesothelioma, uveal melanoma, and various other malignancies. Herein, we revealed the tumor promoting function of BAP1in prostate cancer cells. Intriguingly, some cancer-related genes are characterized to have contrasting functions (tumor suppressor as well as tumor promoter) (Stepanenko *et al.*, 2013; Shen *et al.*, 2018). For instance, nuclear factor 1B (NF1B)—a transcription factor required for the regulation of cell differentiation—functions as both a tumor suppressor (non-small cell lung cancer and osteosarcoma) as well as an oncogene (small cell lung cancer and melanoma) (Becker-Santos *et al.*, 2017). These contradictory functions may be dependent on the cellular context.

Prostate cancer is one of the most common malignant tumors throughout the world. Although the treatment of prostate cancer has improved during the past decade, advanced stages of the disease are still hard to manage. The TCGA dataset analysis suggested that high BAP1 expression is inversely correlated with disease-free survival in the context of prostate cancers. Thus, identification of the mechanisms underlying the effect of BAP1 on the development of prostate cancer will provide valuable insights for the treatment of prostate cancer. BAP1 functions in the regulation of tumor progression have been largely related to its deubiquitinating activity. A number of different BAP1 substrates have been discovered to elucidate the mechanisms by which BAP1 contributes to tumorigenesis (Wang *et al.*, 2016). Histone H2A (Lys119) is a target of BAP1 (Scheuermann *et al.*, 2010). Recently, it has been reported that increase in H2AK119ub on *Snail* in response to the loss of *BAP1* inhibited the transcription of *Snail*, and thus lead to the induction of MET in clear cell renal cell carcinoma (Chen *et al.*, 2019). In the present study, we also confirmed the downregulation of *Snail* in prostate cancer cells upon BAP1 knockdown. It may be possible that BAP1-mediated regulation of EMT—which enhances the invasive and migratory potential in prostate cancer cells—is achieved through its deubiquitinating activity towards H2AK119ub. Nonetheless, as the roles of BAP1 in the context of cancer progression are diverse and context-dependent, it is imperative to find a specific substrate of BAP1—that is involved in the promotion of prostate cancer—to precisely characterize the functional mechanisms of BAP1.

## Supplementary material

The following online material is available for this article:

Figure S1 The expression of apoptotic genes is not affected upon knockdown of BAP1 in prostate cancer cells.

Table S1 Primers used for apoptotic genes in real-time RT-PCR
