# Supplementary material for: BAP1 functions as a tumor promoter in prostate cancer cells through EMT regulation
Source: Genet Mol Biol. 2020 May 8;43(2):e20190328. doi: 10.1590/1678-4685-GMB-2019-0328 (PMC7323895; doi:10.1590/1678-4685-GMB-2019-0328)
Supplement: Figure S1 [file 1415-4757-GMB-43-2-e20190328-s1.pdf]

Supplementary Material to “BAP1 functions as a tumor promoter in prostate cancer cells through EMT regulation”

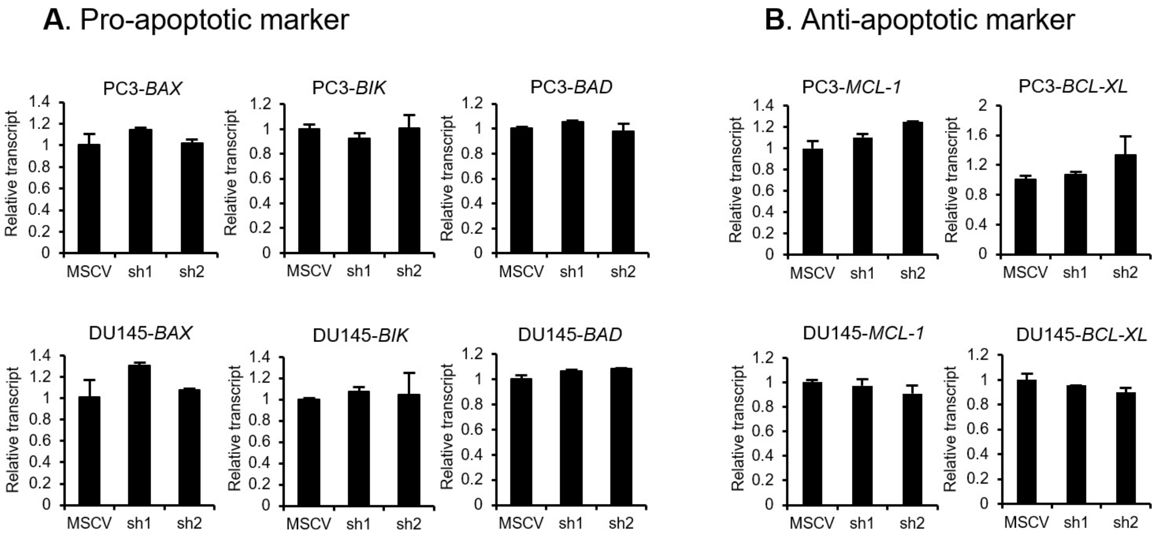

Figure S1 - The expression of apoptotic genes is not affected upon knockdown of BAP1 in prostate cancer cells. (A) Real-time quantitative RT-PCR analysis of pro-apoptotic genes in BAP1-knockdown stable cells. Values are expressed as mean ± SD of three independent experiments. (B) Real-time quantitative RT-PCR analysis of anti-apoptotic genes in BAP1-knockdown stable cells. Values are expressed as mean ± SD of three independent experiments.
