# Supplementary material for: BAP1 functions as a tumor promoter in prostate cancer cells through EMT regulation
Source: Genet Mol Biol. 2020 May 8;43(2):e20190328. doi: 10.1590/1678-4685-GMB-2019-0328 (PMC7323895; doi:10.1590/1678-4685-GMB-2019-0328)
Supplement: Table S1 [file 1415-4757-GMB-43-2-e20190328-s2.pdf]

Supplementary Material to "BAP1 functions as a tumor promoter in prostate cancer cells through EMT regulation"

Supplementary Table 1. Primers used for apoptotic genes in real-time RT-PCR. For pro-apoptotic markers BAK, BIK, and BAD were used and for anti-apoptotic markers MCL-1 and BCL-XL were used.

Gene	Sequence	Reference
BAK	F: TTTACCGCCATCAGCAACCT<br>R: ATAGGCATTCTCTGCCGTGG	Oncotarget. 2017;8(10):17216-17228.
BIK	F: ACCTGGACCCTATGGAGGAC<br>R: GGTGAAACCGTCCATGAAAC	PLoS One. 2009;4(8):e6764.
BAD	F: CCTCAGGCCTATGCAAAA<br>R: AAACCCAAAACTTCCGATGG	Int J Mol Med. 2016;38(1):105-12.
MCL-1	F: AAGAGGCTGGGATGGGTTTG<br>R: CAGCAGCACATTCCTGATGC	Oncotarget. 2017;8(10):17216-17228.
BCL-XL	F: CCTGCCTGCCTTTGCCTAA<br>R: TGGGCTCAACCAGTCCATTG	Oncotarget. 2017;8(10):17216-17228.
